# Editorial: Exploration of cold-adapted microorganisms for sustainable development

**DOI:** 10.3389/fmicb.2023.1191673

**Published:** 2023-04-13

**Authors:** Deep Chandra Suyal, Ajar Nath Yadav, Hesham Ali El Enshasy, Ravindra Soni

**Affiliations:** ^1^Vidyadayini Institute of Science, Management, and Technology, Bhopal, Madhya Pradesh, India; ^2^Department of Biotechnology, Dr. Khem Singh Gill Akal College of Agriculture, Eternal University, Sirmour, Himachal Pradesh, India; ^3^Institute of Bioproduct Development (IBD), Universiti Teknologi Malaysia (UTM), Johor Bahru, Johor, Malaysia; ^4^Faculty of Chemical and Energy Engineering, Universiti Teknologi Malaysia (UTM), Johor Bahru, Johor, Malaysia; ^5^City of Scientific Research and Technology Applications (SRTA), New Borg El-Arab, Alexandria, Egypt; ^6^Department of Agricultural Microbiology, College of Agriculture, Indira Gandhi Krishi Vishwavidyalya, Raipur, Chhattisgarh, India

**Keywords:** agricultural sustainability, cold-adaptation, stress tolerance, cold-active enzymes, microbial diversity

Cold-adapted microorganisms can thrive and colonize every low-temperature habitat available on the Earth, including polar regions, non-polar mountains, and deep-sea environments. They are among the pioneer colonizers of such extreme habitats and may include diverse species of archaea, bacteria, fungi, algae, and other micro-eukaryotes. These microorganisms have shown great ecological, agricultural, and biotechnological potential application for agro-environmental sustainability. They are an excellent source of commercially important antifreeze compounds (Eskandari et al., 2020), cold-active enzymes, cold shock proteins (Mesbah, 2022), and metabolites (Styczynski et al., 2022). Cold-adapted microorganisms have been examined for plant growth promotion, bioremediation, and waste management at low-temperature conditions (Suyal et al., 2022; Kour and Yadav, 2023). Psychrophilic microorganisms have adapted to survive at low-temperature conditions using diverse mechanisms. Several adaptations at molecular and biochemical levels assist psychrophilic and psychrotrophic microorganisms to carry out vital cellular processes under a variety of abiotic stresses prevailing in cold environments (Figure 1).

**Figure 1:**
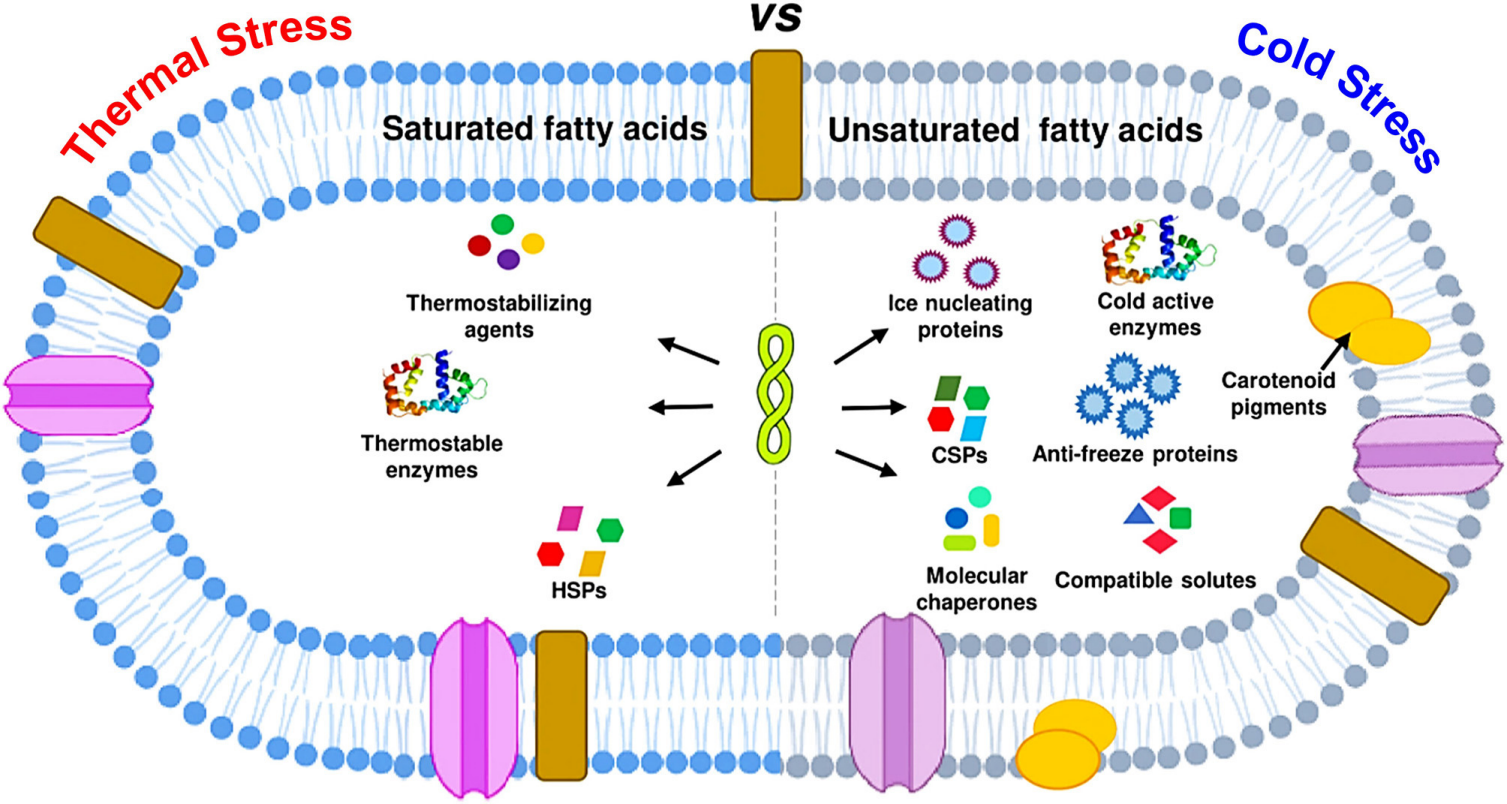
A schematic diagram illustrating some crucial thermal and cold adaptations in cold-adapted microorganisms. Adapted with permission from Suyal et al. (2022).

The cold-adapted agriculturally important microorganisms are “the cost-effective and environmentally friendly alternative of agrochemicals for high-altitude agroecosystems (Rawat et al., 2019; Goel et al., 2022). Among them, *Arthrobacter, Bacillus, Paenibacillus, Pseudomonas*, and *Rhodococcus*, have been identified from these cold habitats (Soni et al., 2015; Joshi et al., 2019). They have shown multifunctional traits including atmospheric nitrogen fixation, phosphorus solubilization, siderophores production, potassium solubilization and mobilization, phytohormone production, and other plant-beneficial activities (Suyal et al., 2022). However, the full potential of cold-adapted microorganisms for agricultural purposes is not been fully explored. Therefore, a detailed investigation of their plant growth promotion abilities, community structure, and temporal as well as spatial field trials is necessary.

This Research Topic includes three original research and one review article on cold-adapted microorganisms. Turchetti et al. have developed a genomic approach to assess and compare the adaptive features of yeasts species associated with the Italian Alps of Alpine and Antarctic regions. It unravels the adaptive strategies of eukaryotic microorganisms under cold conditions *viz*. synthesis of cold-shock proteins, cryoprotectants, and antifreeze compounds; preventing inappropriate protein folding and intracellular ice formation; regulation of gene expression and cellular transport. Further, synchronizing the membrane permeability, electron transport, and nutrient uptake was also employed for increasing survivability. Among proteins, besides synthesizing longer and more hydrophilic loops; the proportion of glycine residues got increased under cold conditions while proline and arginine contents lowered. Furthermore, they have observed several small open reading frames and high genetic redundancy among cold-adapted yeasts that must be considered in future studies.

Ruiz-Blas et al. analyzed the microbial diversity of Pyrenean ice caves in Europe and showed the hidden treasure of potential microorganisms in such unexplored regions. They have used metabarcoding techniques to find the correlation between the physicochemical properties of ice caves with the composition of the microbial diversity. Moreover, they employed a proteomic approach to assess the effect of climate change on the indigenous microbial cells. This study revealed that the microbial distribution in such extreme regions greatly depends upon the age and organic content of the ice. They have also identified specific niches for a few genera. Moreover, they observed that a 4°C rise in the temperature reduced the microbial protein content significantly. It indicates that the effect of climate change on microbial bioactive compounds could be an active area of investigation in near future.

In another article, Dasila et al. evaluated the plant growth-promoting potential of cold-tolerant *Pseudomonas*. They applied four different phosphate-solubilizing *Pseudomonas* strains to the wheat crop under natural field conditions. They observed a significant increase in biochemical and agronomical parameters of the wheat crop with an average grain yield of 22%. Moreover, those strains improved soil health as well. Because of the increasing food demand, food production needs to be increased sufficiently but not at the cost of the environment. Agrochemicals are quicker and specific in action but negatively impact the living world too. At the same time, marginal and uncultivated regions should also be targeted to maximize food production. Therefore, such cold-adapted microbial strains must be utilized for the native crops at high-altitude agroecosystems. It will help in adopting organic and ecofriendly farming practices and thus will contribute to achieving agricultural sustainability goals. A systemic review has been provided by Chauhan et al. on ecological and biotechnological aspects of cold-adapted *Pseudomona*s. Being metabolically diverse, *Pseudomonas* has proven itself as a potential candidate for plant growth promotion, bioremediation, and production of industrially important enzymes as well as bioactive compounds. These all aspects have been summarized in this review along with associated futuristic approaches.

In conclusion, this Research Topic provides useful information and updates on various facets of cold microbiology. We believe, it will lead future studies in this field and will help in achieving sustainable growth and development.

## Author contributions

All authors listed have made a substantial, direct, and intellectual contribution to the work and approved it for publication.
